# Genomics-Based Exploration of Virulence Determinants and Host-Specific Adaptations of *Pseudomonas syringae* Strains Isolated from Grasses

**DOI:** 10.3390/pathogens3010121

**Published:** 2014-01-28

**Authors:** Alexey Dudnik, Robert Dudler

**Affiliations:** Institute of Plant Biology, University of Zurich, Zollikerstrasse 107, 8008 Zurich, Switzerland; E-Mail: alexey.dudnik@botinst.uzh.ch

**Keywords:** *Pseudomonas syringae*, Poaceae, host-pathogen interactions, genomics

## Abstract

The *Pseudomonas syringae* species complex has recently been named the number one plant pathogen, due to its economic and environmental impacts, as well as for its role in scientific research. The bacterium has been repeatedly reported to cause outbreaks on bean, cucumber, stone fruit, kiwi and olive tree, as well as on other crop and non-crop plants. It also serves as a model organism for research on the Type III secretion system (T3SS) and plant-pathogen interactions. While most of the current work on this pathogen is either carried out on one of three model strains found on dicot plants with completely sequenced genomes or on isolates obtained from recent outbreaks, not much is known about strains isolated from grasses (Poaceae). Here, we use comparative genomics in order to identify putative virulence-associated genes and other Poaceae-specific adaptations in several newly available genome sequences of strains isolated from grass species. All strains possess only a small number of known Type III effectors, therefore pointing to the importance of non-Type III secreted virulence factors. The implications of this finding are discussed.

## 1. Introduction

Strains of the *Pseudomonas syringae* group are causal agents of a variety of plant diseases worldwide. Strains of this group have been reported to infect nearly 200 different plant species [1], including both grassy and woody hosts. A number of those are agriculturally important plants, and as a consequence, *P. syringae* is one of the best-studied plant pathogens. While some diseases have been known to recur in the form of outbreaks for a long time [2,3,4,5], others have only recently emerged [6,7]. The taxonomy of *P. syringae* is still under discussion. According to the commonly used version, the species is sub-divided into over 50 groups called “pathovars”. This division takes into account host-specificity, disease type, as well as the biochemical characteristics of a strain [8,9]. In contrast, a number of phylogenetic studies have positioned *P. syringae* as a species complex, and depending on the approach used, it has been split either into five phylogenetic groups by multi-locus sequencing [10,11], or into nine so-called genomospecies based on DNA hybridization profiles [12]. In order to remain coherent with the majority of publications, in this work, we are going to regard *P. syringae* as a single species.

The observed large genetic diversity among different pathovars is a direct consequence of the wide host range. Moreover, heterogeneity is also found among strains belonging to the same pathovar [6,8,13]. The highest degree of variation is seen within the complement of virulence factors, which is the key element determining host range and the overall degree of virulence [14]. In addition to that, some strains possess host-specific metabolic pathways, such as enzymes for lignin degradation in pathogens of woody hosts [6,7,15]. For successful survival and reproduction, both epiphytic and endophytic strains of *P. syringae* deploy different sets of Type III and Type VI secreted effector proteins, exopolymeric substances, phytohormones, phytotoxins and other types of secreted molecules [14,16,17,18,19,20,21,22,23]. Among those, the major pathogenicity factor is the Type III secretion system (T3SS). Other notable virulence factors are the necrosis-inducing phytotoxins, syringomycin and syringopeptin, which are presumed to create pores in plant cells by imbedding into the plasma membrane, and the anti-metabolite toxins, tabtoxin, mangotoxin and phaseolotoxin, which inhibit glutamine synthetase, ornithine acetyltransferase and ornithine carbamoyl transferase, respectively [19,24]. Another group of phytotoxins includes coronatine and syringolin, both of which are involved in the inhibition of the salicylic acid-dependent immune response by mimicking jasmonic acid or by irreversibly inhibiting the proteasome, respectively [18,19].

The T3SS is a protein delivery machinery, which uses a structure, called injectosome, for the delivery of effector proteins directly into host cells by puncturing the cell membrane [25,26]. This machinery is essential for the pathogenesis of *P. syringae*, and knocking it out renders the bacterium avirulent. [14,17] The majority of Type III effectors (T3Es) are assumed to be involved in the suppression of plant defense, including pathogen-associated molecular pattern (PAMP)-triggered immunity and the hypersensitive response (effector-triggered immunity) [27,28]. In addition, some effectors were shown to have a cytotoxic effect [29,30]. Currently, there are 58 verified effector families recognized [31]. With a few exceptions, the exact mechanism of action of T3Es remains unknown. Among the well-characterized T3Es is AvrPtoB, which targets the flagellin recognition receptor, FLS2, and marks it for degradation [32], HopU1, which ADP-ribosylates several RNA-binding proteins, thus preventing the association with their target mRNAs [33], HopN1, which targets photosystem II in order to inhibit reactive oxygen species production [34], AvrRps4, which targets a regulator of plant basal defenses [35], and HopZ1a, which interferes with plant microtubule network formation and jasmonic acid signaling [36,37]. Most *P. syringae* strains produce around two to three dozen T3Es [6,8,31]. However, not all of them are essential for full virulence, due to functional redundancy [38]. As a consequence, strains pathogenic to the same host often have divergent sets of effectors [6,8,13,39]. It is notable that effector repertoires are under heavy evolutionary pressure [27] and, thus, are being continuously remodeled. The remodeling might also result in a change of host specificity. The field of effector biology remains a hot topic, and currently, a lot of research is aimed at improving our knowledge about the molecular biology of interactions between plants and their pathogens.

The extensive research has also led to the accumulation of a large amount of available genome sequence data. Currently, GenBank contains records of the three completely sequenced *P. syringae* model strains DC3000 (pathovar *tomato*, *Pto*; pathogenic to tomato, *Arabidopsis thaliana* and *Nicotiana benthamiana* [40,41]), B728a (pathovar *syringae*, *Psy*; the causal agent of brown spot disease of bean [42]) and 1448A (pathovar *phaseolicola*, *Pph*; causes halo blight on bean [43]). The strains represent phylogenetic Clades one, two and three, respectively [11,31]. In addition, a number of incomplete genome sequences of various qualities are available for a variety of strains [6,7,8,15,31]. The majority of the sequenced strains were originally isolated from dicot plants; thus, the topic of diversity and adaptations of strains pathogenic to monocot plants remains largely unexplored. Currently, nine genome sequences of different qualities of strains isolated from true grasses (graminoids, family Poaceae) are available. These encompass four wheat (*Triticum aestivum*) isolates (*P. syringae* pv. *syringae* (*Psy*) strains SM and B64, *P. syringae* pv. *atrofaciens* DSM50255 (*Paf*) and *P. syringae* BRIP39023 [44,45,46]), three strains pathogenic to barley (*Hordeum vulgare*) (*P. syringae* BRIP34876 and BRIP34881 [46] and pathovar *japonica* strain M301072 (*Pja*) [31]), one strain collected from rice (*Oryza sativa*) (pathovar *oryzae* 1_6 (*Por*) [47]) and an isolate pathogenic to proso millet (*Panicum miliaceum*) (pathovar *panici* strain LMG2367 (*Ppa*) [48]). Here, we analyze these nine genomes using comparative genomics tools with the aim of identifying possible adaptations of *P. syringae* strains to life in graminoid host species. In addition, we also compared these strains with a group of unspecialized pathogenic strains belonging to *P. cannabina* pv. *alisalensis* (*Pcal*), which have been shown to colonize several dicot hosts, as well as oat (*Avena sativa*) and great brome (*Bromus diandrus*) plants, both of which belong to the Poaceae [49].

## 2. Results and Discussion

### 2.1. Phylogenetic Assessment of the Strains

**Figure 1:**
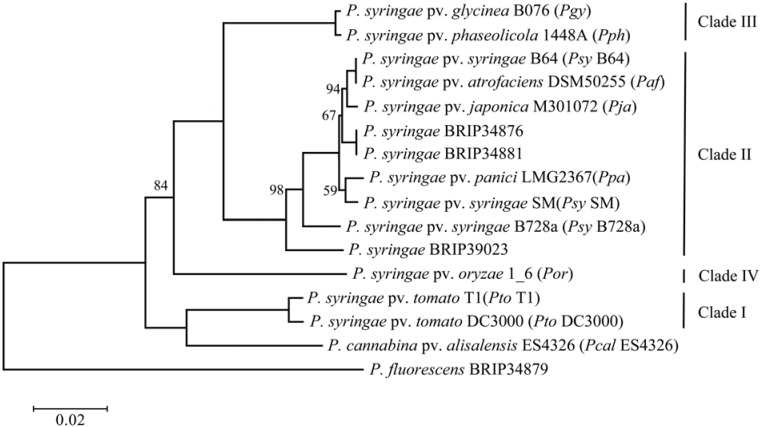
Maximum likelihood phylogenetic tree of the analyzed *P. syringae* strains. *Pgy, P. syringae* pv. *glycinea; Pph, P. syringae* pv*. phaseolicola*; *Psy*, *P. syringae* pv. *syringae*; *Paf*, *P. syringae* pv. *atrofaciens*; *Pja*, *P. syringae* pv. *japonica*; *Ppa*, *P. syringae* pv. *panici*; *Por*, *P. syringae* pv. *oryzae*; *Pto*, *P. syringae* pv. *tomato*; *P. cannabina* pv. *alisalensis* (*Pcal*).

Since most of the analyzed genomes have been sequenced within the past year, there is no published record of their phylogenetic characterization. Thus, the first step undertaken was to identify their relatedness by constructing a maximum likelihood tree using MLST (Multi-locus sequence typing) loci previously deployed in other studies [10,31]. Several other strains belonging to the three major phylogenetic clades [11] have been included as a reference. *P. fluorescens* BRIP34879, a strain which was also isolated from barley [46], was used as an outgroup. *Pcal* ES4326 (previously known as *P. syringae* pv. *maculicola* ES4326) was included to represent the phylogenetic relatedness of the *P. cannabina* pv. *alisalensis* group. The resulting diagram is presented in Figure 1. Even though whole-genome-based phylogenies were recently demonstrated to be more accurate than MLST [50], we still decided to use the MLST approach, due to the poor assembly quality of some of the analyzed genomes, which resulted in a large number of partial and split genes. The presence of such sequences would introduce a bias to the analysis using the whole genome/proteome sequences.

The tree was constructed based on the nucleotide sequences of several housekeeping genes. The scale bar indicates the number of amino acid changes per site. Bootstrapping-based probabilities are displayed only at nodes where the values are less than 100.

While most of the *P. syringae* strains that are the focus of this article cluster together within Clade II, *Por* 1_6 clearly forms a separate branch. This is in line with previously published data, where this strain was placed into Clade IV (also classified as *Pseudomonas coronafaciens* by Gardan and colleagues [12]). Interestingly, Clade IV contains several other strains isolated from cereal crops, including oat [11]. This suggests that a host-shift towards Poaceae has occurred at least twice in the evolution of *P. syringae*. Within Clade II, strain BRIP39023, a non-pathogenic wheat isolate, forms a separate branch and, therefore, is also less related to the rest of the isolates. The remaining strains form a cluster and, therefore, must be of the same ancestral lineage. It is notable that pathovars *syringae*, *atrofaciens*, *panici* and *japonica* do not form distinct branches. This shows that discrimination into pathovars is not always descriptive, since particular strains often are pathogenic to several hosts, as shown, for example, for strains BRIP34876, BRIP34881 and *Ppa* [46,48]. This also suggests that a pathogen can relatively easily change its specificity from one host to another among related plant species. Finally, it is not clear to which pathovar BRIP34876 and BRIP34881 should be assigned. It should be noted that there are several other *P. syringae* pathovars belonging to Clade II (*lapsa*) and Clade IV isolated from Poaceae [12,51], which were not included in this study, due to the lack of available genomic sequence data. *Pcal* ES4326 forms a distinct branch from *Pto* strains and, together with several related strains, was classified as Clade V by Hwang and colleagues [11]. The resulting tree is in agreement with previously published data [11,31].

### 2.2. Genome Comparison and Identification of Poaceae-Specific Genes

During the process of adaptation to a new host, there is a strong evolutionary pressure on a pathogen. As a consequence, a pathogen loses some genes that reduce the virulence and overall fitness in the new host. In addition, novel genes are acquired by horizontal gene transfer (HGT), in particular from species already living on or inside the new host. Examples include genes for the degradation of lignin, pectin and aromatic compounds found in *P. syringae* strains pathogenic to woody hosts [15,52]. Therefore, the strains isolated from grasses could also exhibit certain adaptations not found in other strains. In order to identify candidate adaptation genes, the genomes of the nine sequenced Poaceae isolates were first compared among themselves and, then, also, with a group of twelve other *P. syringae* strains isolated from a variety of hosts (See Table S1 for a complete list of strains). The comparison was performed using an 80% identity cut off in order to avoid the detection of paralogs and other genes with only partial homology. An outline of the results is shown in Table 1, and a list of the identified unique genes, which are shared by at least four strains, can be found in Table S2.

**Table 1:** Genome comparison of the nine analyzed Poaceae isolates: unique genes.

Ortholog clusters	A (within grass isolates)	B (not found outside grass isolates)
Unique to *Por*	2,333	1,566
Unique to BRIP34876	11	8
Unique to BRIP34881	13	7
Unique to BRIP39023	349	121
Unique to *Pja*	3,657	2,563
Unique to *Psy* SM	178	75
Unique to *Psy* B64	37	9
Unique to *Ppa*	484	322
Unique to *Paf*	216	188

Column **A** represents the numbers of ortholog clusters obtained by comparing the nine genomes of Poaceae isolates among themselves. Column **B** contains an overview on the distribution of ortholog clusters found exclusively in these nine isolates, but not outside. An ortholog cluster is a group of genes from at least one strain in which all members have an identity percentage equal to or above the set cut off. Thus, a single cluster might contain more than one gene per strain, due to the presence of recent gene duplications, which are still more than 80% identical to one another. Such genes are regarded as the same entity by the software, and as a consequence, the actual numbers of shared homologs are slightly different for each strain.

**Figure 2:**
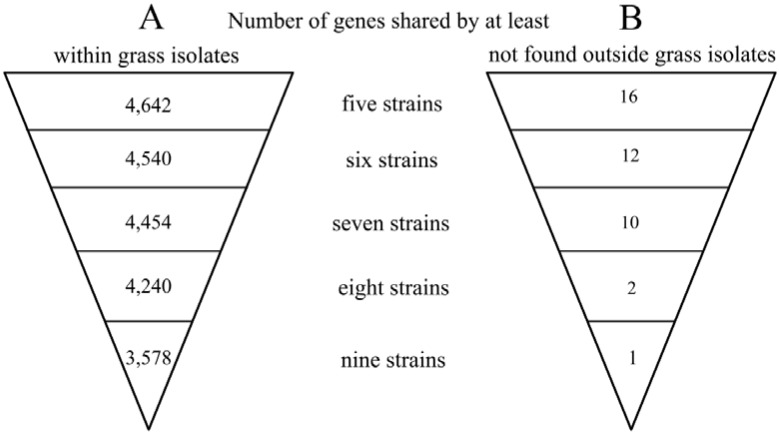
Shared genes in the genomes of the nine analyzed Poaceae isolates.

When the Poaceae isolates were compared to one another, the total number of identified ortholog clusters was 13,319. The calculated core genome, *i.e.,* the set of genes shared by all strains, has a size of 3,578 ortholog clusters (Figure 2A), which is comparable to the previously published data [31]. Outside the core genome, 2,471 clusters are shared by at least two strains. An overview of shared genes is depicted in Figure 2A. The remaining ortholog clusters have only one member, and respective genes are therefore unique to one of the strains. The number of unique genes is highly variable, ranging from as little as eleven for BRIP34876 to almost 3,657 for *Pja* (Table 1, Column A). The large values observed for *Pja* and *Por*, however, originate from the low quality of the respective assemblies (see Table S1). Because of this, there are many proteins that are found on several contigs. For example, the *Pja* genome contains seven entries for the chromosomal replication initiation protein, DnaA, which is normally present as a single copy next to the origin of replication. It is highly likely that many partial sequences did not pass our strict identity criterion and therefore ended up as individual ortholog clusters. Moreover, the majority of the genomes did not undergo manual curation, and thus, their annotation data contains a number of very small hypothetical, as well as truncated proteins.

Column **A** represents the numbers of ortholog clusters shared by five or more of the nine Poaceae isolates when compared among themselves. Column **B** contains an overview of the distribution of ortholog clusters found exclusively in these isolates, *i.e.*, ortholog clusters that were not detected in any other analyzed *P. syringae* strain.

Surprisingly, however, there is only a single gene that is found in all nine Poaceae isolates, but is absent in any of the other *P. syringae* genomes included in the comparison (Figure 2B and Table S2; see Section 2.4.2). Furthermore, the additional gene shared by eight strains (all except BRIP39023) encodes a methyltransferase, which has no homologs in any other sequenced genome. Moreover, there are only 36 additional genes that are found in at least four of the Poaceae-colonizing strains (Figure 2B and Table S2). Out of these 36, only four are present in the *Por* genome, which is most likely due to the different phylogenetic lineage of this strain. Interestingly, most of these genes are found within four genomic regions that are flanked by sequences with different G + C profiles and transposable elements (as marked in Table S2). Regions 1 and 2 are present in all of the Clade II strains, except BRIP39023, which makes it very tempting to speculate that the asymptomatic phenotype of this strain could be, at least partially, due to the absence of some of these genes. This is not completely unjustifiable, as these genomic regions encode several proteins that could potentially be involved in the protection from oxidative stress and anti-microbial compounds. These include a catalase-like protein, cytochrome b561 and a small multidrug resistance protein. Additionally, there are also three transcriptional regulators that might be needed for the fine-tuning of gene regulation. The other two genomic regions are present in only four strains, but aside from hypothetical proteins, they encode a putative formaldehyde dehydrogenase, a putative general stress protein, a sensor histidine kinase and an MFS (Major Facilitator Superfamily) transporter. However, this conclusion is purely speculative, and the characterization of deletion mutants of these regions is required to elucidate their function.

### 2.3. The Type III Secretion System and Effector Repertoire

**Figure 3:**
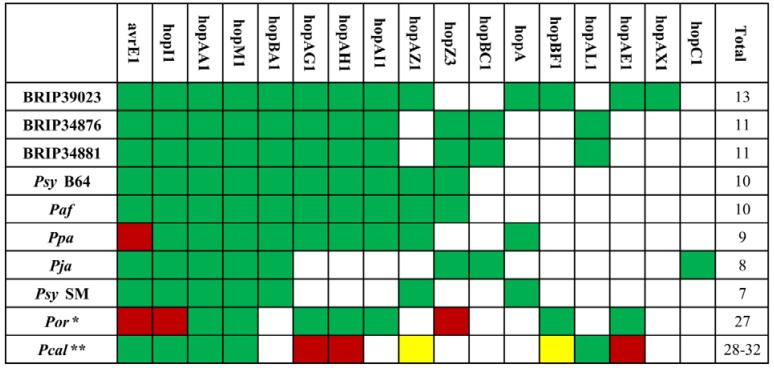
Type III effector repertoires.

Based on genome sequences, the analyzed strains possess the canonical *hrp*/*hrc*-type type III secretion system [26,53], which is found in most of the sequenced *P. syringae* isolates. The effector repertoires for each strain are presented in Figure 3. Based on the number of T3Es, the strains form two distinct groups: the first one consists of *Por*, which has 27 full-length T3Es, while the remaining eight strains only contain around a dozen effectors. The distribution also correlates with the phylogenetic relatedness of the strains (see Figure 1), further supporting the assumption that the host shift has occurred independently for the two groups. Moreover, *Por* has only five effectors shared with *Ppa*, which was also reported to infect rice [48], therefore supporting the idea that the same host can be colonized by strains with very little overlap in effector composition. Interestingly, both strains lack AvrE1. Unfortunately, however, there are no reliable data on T3Es from other rice isolates to further validate these findings.

Apart from *Por*, the remaining eight *P. syringae* strains contain a clear core effector set consisting of AvrE1, HopI1, HopAA1, HopM1 and HopBA1, of which the first four are found in intact form in most sequenced *P. syringae* strains [6,31]. The phylogenetic relationship of the core T3Es is shown in Figure S1. The presented individual phylogenetic trees in general reflect the one obtained using the MLST approach (Figure 1). Interestingly, HopBA1 is identical in nearly all strains, suggesting a strong evolutionary pressure to maintain the sequence. Moreover, the remaining effectors appear to have different substitution rates, as seen from the corresponding scale bars. The other most common effectors are HopZ3, HopAZ1 and the HopAG1-HopAH1-HopAI1 cluster. The effector repertoire also correlates with phylogenetic data, as, for example, *Ppa* differs from its closest relative, *Psy* SM, only by the HopAG1-HopAH1-HopAI1 locus. Interestingly, the avirulent strain, BRIP39023, has the largest number of effectors, implying that the number of effectors does not correspond to disease severity and overall virulence. However, it is also possible that one of the effectors acts as an avirulence (Avr) protein, triggering a local hypersensitive response and, therefore, suppressing endophytic growth. 

All five currently sequenced *Pcal* strains contain a relatively large number of T3Es, which includes 28 to 32 full-length, as well as five to eleven truncated or disrupted effectors [49]. Out of those, 16 full-length T3Es are shared with *Por*: AvrPto1, HopAA1, HopAB3, HopAD1, HopAO1, HopAQ1, HopAS1, HopBF1, HopD1, HopG1, HopM1, HopQ1, HopR1, HopV1, HopBI1 and HopX1. So far, all *P. syringae* strains isolated from oat that were used in scientific studies belong to Clade IV [11,54] and, thus, are likely to have comparable effector repertoires. Assuming that this is true and applies also to the *P. syringae* pv. *coronafaciens* (Clade IV) strains, one might expect that they might be able to infect both monocots and dicots, too. However, these strains were reported to cause disease symptoms on brome, rye and oat, but not on crucifers or tomato [55]. Interestingly, neither *Pcal* nor pathovar *coronafaciens* strains were able to cause disease in wheat [56]. In contrast to *Por*, *Pcal* strains have very little overlap with the Clade II *P. syringae* strains with regard to their T3E repertoire. In addition to the core effectors, AvrE1, HopAA1, HopM1 and HopI1, only HopAL1, HopAZ1 and HopBF1 are found in some *Pcal* strains.

Green squares indicate the presence and blank squares indicate the absence of the respective T3Es, and red squares indicate a truncated or disrupted effector. Yellow indicates that an effector is not present in all strains assigned to this species. The total number of effectors is indicated at the end of each row.

* The genome of *Por* contains an additional 20 full-length effector-coding genes not found in any of the eight other analyzed *P. syringae* strains (*hopAQ1*, *avrB1*, *avrB3*, *avrPto1*, *avrPto4*, *hopAD1*, *hopAS1*, *hopX1*, *hopAO1*, *hopD1*, *hopG1*, *hopR1*, *hopV1*, *hopY1*, *hopAB3*, *hopH2*, *hopQ1-1*, *hopQ1-2*, *hopBH1* and *hopBI1*).

** Likewise, for the *Pcal* strains, only effectors that are also present in Clade II strains are shown. For a detailed T3E composition of the individual *Pcal* strains, see [49]. It should also be note that some of the genomes of these strains encode a number of the so-called discontinued T3Es, which were never experimentally shown to be translocated in a T3SS-dependent manner: HopAN1, HopAC1, HopJ1, HopL1 and HopAH2.

Having relatively few T3Es is not uncommon for *P. syringae* and seems to be a feature of Clade II strains [31]. However, the strains isolated from Poaceae species are rather extreme examples, and at present, it remains unclear why this is the case. Some T3Es form so-called Redundant Effector Groups (REGs) [38], where members of a REG target different steps in a particular defense signaling pathway. Thus, effectors within one REG are often interchangeable, and the possession of one member of each of the required REGs should be sufficient for full virulence. In support of this notion, the minimal effector repertoire for *Pto* DC3000 on *A. thaliana* has been identified to consist of eight out of 31 T3Es [38], a value which is similar to what is seen in the Poaceae-colonizing strains. Therefore, it is possible that in these hosts, there is a strong selection pressure to maintain just one effector for each REG. On the other hand, there is always the possibility that these genomes contain a number of unrecognized novel T3Es. However, based on results by Baltrus and colleagues [31], there should be very few undiscovered effector families, and it is therefore unlikely that our search missed a considerable number of unknown T3Es.

In addition to the *hrp*/*hrc*-type T3SS, several *P. syringae* strains were described to possess a second rhizobial-like T3SS, belonging to the *Rhc* family subgroup II [57]. This T3SS-2 was originally identified in *Pph*, *Pta* and *Por*. In *Por*, the gene cluster encompasses 31 genes (POR16_18228–POR16_18383) and appears to contain all essential structural components [57]. Among the other Poaceae isolates, we have identified another T3SS-2 gene cluster in BRIP39023 (A988_16153–A988_16283, accession no. KB316298). Structure-wise, the gene cluster rather resembles its homolog in *Pta* than that of *Por* or *Pph*. It is not, however, known whether this T3SS is active and plays any role in interactions with the host. Moreover, the genomes of both BRIP39023 and *Por* do not contain any known rhizobial effectors, based on sequences from *Rhizobium*, *Sinorhizobium* and *Bradyrhizobium* deposited on the T3DB (Type-III-Secretion-System Related Database) website [58].

### 2.4. Other Virulence Factors

#### 2.4.1. Phytotoxins and Other Small Secreted Molecules

While the major pathogenicity determinant is the T3SS, many *P. syringae* strains also rely on other secreted molecules for entry and colonization of their hosts. The most important and best-studied group of such compounds are the phytotoxins. There seems to be no general requirement for phytotoxin production, and while some strains are capable of producing several different compounds, others lack any known phytotoxins [19,31]. Moreover, even strains belonging to the same pathovar have differences in phytotoxin gene composition [8,59,60]. Nevertheless, it is a well-established fact that their presence enhances disease progression and symptom development [19,61,62]. In terms of overall distribution, syringolin, syringomycin, syringopeptin and mangotoxin are found almost exclusively among Clade II strains, while all other phytotoxins are found outside of this phylogenetic group [31,63]. So far, the only described exception is *P. syringae* pv. *syringae* CFBP3388, a Clade II strain that was identified to produce both phaseolotoxin and mangotoxin [60,63].

**Figure 4:**
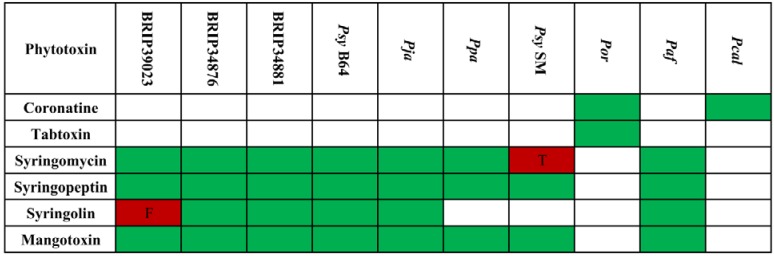
Distribution of phytotoxins among the analyzed strains.

The composition of phytotoxin biosynthetic gene clusters among the Poaceae isolates (Figure 4) fits into the above-mentioned phylogenetic clade-dependent distribution scheme. The genome of *Por*, which belongs to Clade IV, contains gene clusters for the biosynthesis of coronatine and tabtoxin, whereas all other strains have genes for the production of mangotoxin, syringopeptin and syringomycin. However, *Psy* SM seems unable to produce syringomycin, as its *syrE* gene is truncated. It should be noted that the biosynthetic gene clusters for syringopeptin and syringomycin encode very large non-ribosomal peptide synthetases (NRPS) containing repetitive sequences. Because these clusters are often found on several contigs, their complete intactness cannot be assessed with certainty. Complete and intact syringolin synthetase gene clusters, and, thus, the ability for syringolin production, are present in all Clade II strains analyzed, except in *Psy* SM, *Ppa* and BRIP39023. The first two do not have a syringolin synthetase gene cluster, whereas the latter strain contains a frameshift mutation in the *sylA* gene. Genomes of the *Pcal* strains only contain coronatine biosynthesis genes. None of the analyzed strains is capable of producing phaseolotoxin. In conclusion, there appears to be no specific requirement in terms of toxin composition for being able to colonize Poaceae species.

Some *P. syringae* strains are known to produce phytohormones, such as the auxin indole 3-acetic acid (IAA), cytokines and ethylene [64,65,66]. While cytokine biosynthesis appears to be specific for *P. syringae* pv. *savastanoi* and ethylene production was detected only in pathovars *pisi*, *cannabina*, *glycinea*, *phaseolicola* and *sesame*, IAA biosynthetic genes appear to be widespread among all major phylogroups [31,66]. Interestingly, none of the Poaceae isolates have homologs of the characterized tryptophan 2-monooxygenase (*iaaM*) and indoleacetamide hydrolase (*iaaH*) genes from *P. syringae* pv. *savastanoi* (*Psv*) NCPPB3335 (accession M11035). However, there appears to be an alternative pathway for IAA biosynthesis, as there are a number of strains where IAA production was demonstrated experimentally, but which lack homologs of the *iaaM^Psv^*/*iaaH^Psv^* genes [67]. The majority of *P. syringae* genomes, including the nine Poaceae isolates, encode another gene annotated as tryptophan 2-monooxygenase at a different genomic location (see accession AAY39694 as a reference). There is also an R-amidase-like protein encoded 15 bp downstream of this gene, and most likely, these two genes constitute an operon. Considering the fact that IaaH also belongs to the amidase family, it is possible that this putative operon is responsible for IAA production in strains lacking the *iaaM^Psv^*/*iaaH^Psv^* homologs. A similar situation is observed for the *Pcal* strains. Apart from that, N-ε-(indole-3-acetyl)-L-lysine synthetase (*iaaL*), which modifies IAA, is present in both the *Por* and *Pcal* strains [68]. The ethylene biosynthesis gene, *efe*, is present only in the genomes of the *Pcal* strains, but not in the Poaceae isolates.

Green squares indicate the presence, while blank squares indicate the absence of the respective biosynthetic genes. “F” indicates a frameshift in at least one gene, “T” indicates that at least one gene is truncated.

#### 2.4.2. Quorum Sensing

Bacterial cell-to-cell communication is an important part of bacterial physiology that influences many processes, such as growth, differentiation and virulence [69]. One type of such intercellular communication is dependent on cell density and referred to as quorum sensing (QS). The first described QS system is regulating luminescence in *Vibrio fischeri* [70], and ever since its discovery, QS research continues to be a hot topic, due to its large spectrum of influence. A typical Gram-negative type QS system consists of two proteins encoded by adjacent genes: a LuxI-family synthetase, which produces a signaling compound (in the vast majority of cases, these are acyl-homoserine lactones, AHL) and a LuxR-family transcriptional regulator. Upon perception of the respective AHL, the LuxR protein activates transcription of *luxI*, as well as of other genes that contain a *lux*-type box in their promoters [69]. QS has been show shown to be important for a number of plant pathogens, including *Agrobacterium tumefaciens*, *Pantoea stewartii*, *Xanthomonas campestris* and *Erwinia carotovora*, where it regulates processes, such as Ti plasmid conjugation, secondary metabolite production, enzyme and exopolysaccharide (EPS) secretion, *etc.* [71]. Many *P. syringae* strains also possess a QS system encoded by the *ahlR*-*ahlI* locus [72], which was demonstrated to be involved in the regulation of epiphytic fitness [71].

Interestingly, none of the analyzed Poaceae-colonizing strains, as well as none of the currently sequenced *Pcal* strains contain *ahlR*-*ahlI* homologs or any other known quorum sensing system found in Gram-negative bacteria. The trend seems to be general for strains isolated from Poaceae and also extends to pathovars *atrofacies* and *lapsa* [51]. Moreover, strain *Ppa* CFBP 2345 used in that study and *Ppa* LMG2367 are identical [73], thus validating our genomics-based findings. Because of the demonstrated importance of the QS for a number of pathogens, its loss might be expected to have negative consequences on leaf colonization. However, since the lack of the canonical QS system is relatively abundant among *P. syringae* group strains [51], it is likely that the AHL-mediated gene regulation has no significant effect on the T3SS in these strains. As to why the lack of QS appears to be a general pattern for Poaceae isolates, this could be connected to the fact that some groups of AHLs are actually recognized by plant cells and induce changes in gene expression, including defense genes [74]. Moreover, exposure to AHL was shown to be an inducer of systemic acquired resistance in barley [75]. The AHL type used in that study was different from the one produced by *P. syringae*. However, it is likely that the recognition spectrum is broader, thus making AHL producers less virulent and giving a selective advantage to those which have lost the respective genes.

Bacterial genomes often encode orphan LuxR-type transcriptional regulators, which are not associated with a cognate LuxI partner. Some were shown to bind signaling molecules originating not only from cells of the same species, but also coming from other bacteria and even from eukaryotic organisms [74,76,77]. There also exists a distinct clade of the orphan LuxR proteins identified in several groups of plant-associated bacteria, which were reported to have an affinity towards plant-borne low-molecular weight compounds [78]. For example, the LuxR family member OryR from *X. oryzae* pv. *oryzae* was identified to be a global regulator and a virulence factor that responds to the presence of homogenized plant material, but not to AHLs [79,80]. With the exception of BRIP39023, all other Clade II Poaceae isolates have a gene encoding an orphan LuxR family protein that is not found in any other *P. syringae* genome (see Table S2). This gives room for speculation that this protein could be involved in the detection of plant signals that are associated with Poaceae. However, unlike other such sensor proteins, it is not encoded adjacent to a proline immunopeptidase *pip* [74]. The protein shares 78% sequence similarity with the syringomycin biosynthesis regulator, SyrG, of *Psy* B301D [81]. Syringomycin production is known to be enhanced by p-arbutin [82], which is a glycosylated hydroquinone found in plant tissues and which also appears to be relatively abundant in wheat [83]. It is not known whether SyrG actually binds this compound; however, it seems possible that these proteins are able to interact with plant-borne phenolic compounds.

Additionally, the only identified unique gene that is shared among all nine genomes belongs to the LysE/RhtB family of efflux proteins, which are hypothesized to be involved in the export of AHLs [84]. One proposed function for this family of exporters is protection from interference by AHLs produced from other bacterial species. Interestingly, two recent studies showed the production of AHL-mimicking substances in rice and *Medicago truncatula* [85,86], and therefore, it is possible that such a protein could be involved in the protection of the bacteria from these compounds.

#### 2.4.3. Exopolysaccharides

*P. syringae* strains are known to produce two types of EPS: levan and alginate [87]. While levan is a branched β-polyfructan [87], alginate consists of blocks of non-repeating α-L-glucuronate and β-D-mannuronate, with the latter residue being randomly acetylated [88]. Levan is produced from sucrose by the action of a single enzyme, called levansucrase [89]. *P. syringae* genomes usually contain more than one levansucrase gene located at different genomic sites [89]. In contrast, alginate biosynthesis is a multi-step process, which involves several enzymes [88]. Respective genes are organized in a gene cluster and have an identical architecture in both *P. syringae* and *P. aeruginosa* [90]. One of the major roles of EPS is thought to be protection from desiccation. Furthermore, while alginate was shown to be involved in epiphytic fitness and osmotolerance [22,90], levan is presumed to be a storage polymer [91]. In addition, most *P. syringae* strains have homologs of genes from *P. aeruginosa* involved in the biosynthesis of another EPS called Psl. Psl, a complex polysaccharide with repeating pentamer units of D-glucose, D-mannose and L-rhamnose [92], was demonstrated to be required for biofilm formation in *P. aeruginosa* [93].

When compared with one another, all Poaceae isolates possess the alginate biosynthesis cluster (*algD*-*algA*), as well as all other genes involved in its biosynthesis and regulation (*algP*, *algQ*, *algR*, *algZ*, *algC*, *algB*, *kinB*, *algH*, *algU*, *mucA*, *mucB* and *mucD*). With respect to levan production, all strains were identified to have the *lscA* gene, which, however, was shown not to contribute to levan production [89]. With the exception of *Por*, all other genomes contained a copy of the *lscC* gene. In *Paf*, both *lscA* and *lscC* are located on two separate contigs, and thus, it is not possible to tell whether the two genes are completely intact. None of the strains had a plasmid-born *lscB* gene. Thus, among the analyzed strains, only *Por* appears to be levan-negative. Last, with respect to *psl* gene content, *P. syringae* is slightly different from *P. aeruginosa* and lacks a homolog of the acyltransferase *pslL*. In addition, the *pslC* gene is located outside of the main gene cluster. However, all nine genomes contain an additional gene between the *pslJ* and *pslK* homologs, which encodes a putative maltose O-acyltransferase family protein. Since the *psl* gene cluster was shown to be transcriptionally active [94], it is possible that *P. syringae* is producing a slightly different version of this EPS. The analyzed *Pcal* strains appear to contain genes for the biosynthesis of both alginate and Psl. A single levansucrase-coding gene is present in all of these strains, with the exception of ES4326, which did not give any hits in our BLAST (Basic Local Alignment Search Tool) search.

#### 2.4.4. Type VI Secretion System

The Type VI secretion system (T6SS) is a multipurpose protein delivery machinery encoded as a single gene cluster [95]. It is found in the genomes of Gram-negative bacteria, both pathogenic and non-pathogenic, with the highest abundance among Proteobacteria species [95,96]. It is not uncommon that a single genome contains more than one T6SS gene cluster [96,97]. However, different T6SS are found within one genome function independently, and there is no overlap in terms of the translocated effector repertoire [98,99]. In several species, the T6SS was identified to be a pathogenicity factor or at least a virulence enhancer. However, its function is not limited to that, and it was also shown to be involved in inter-species competition, protection from predation and biofilm formation [96]. The first identified effectors for this secretion system were Hcp and VgrG family proteins, which are also structural components of the injection apparatus and are encoded within and outside the T6SS gene cluster [96]. A recent study on *Pto* DC3000 T6SS gene clusters (or HSI, Hcp secretion islands) showed that the Hcp-2 protein encoded in the HSI-2 gene cluster plays a role in competition with other bacterial species, but not in virulence [16].

All nine genomes of Poaceae-colonizing strains contain two individual T6SS gene clusters, which belong to the Group 1 and Group 4B loci based on the homology of the respective ClpV proteins, as classified by Barret and colleagues [97]. However, the *tssB* gene in the T6SS-2 gene cluster (a Group 4B member) of *Pja* contains a potential frameshift. Both types of T6SS are found in most other sequenced *P. syringae* strains [97] and, at least in the case of Poaceae isolates, are located within the same genomic regions. Thus, it appears that these T6SS gene clusters were acquired long ago. A detailed analysis of the T6SS gene clusters from plant-pathogenic bacteria has been performed by Sarris and colleagues [100,101], which also depicts the phylogenetic position of both T6SS-1 and T6SS-2 from *Por* and, thus, of their respective homologs from the other analyzed Poaceae isolates. It is notable that none of the genomes encodes a homolog of the above-mentioned Hcp-2 from *Pto* DC3000.

While the HSIs remain conserved, the number of putative orphan effectors varies between the strains. For example, the genome of *Psy* SM encodes five orphan Hcp and three orphan VgrG proteins; *Psy* B64 has only two such Hcp and three such VgrG-coding genes; whereas the genome of *Ppa* encodes for three members of the Hcp family and five VgrG family members located outside the HSIs. The orphan VgrG family proteins sometimes carry an additional domain at their C-terminus and are referred to as “evolved” VgrG proteins. Some of these domains have been described to have a catalytic function, such as actin crosslinking [96,102]. Among the analyzed genomes, two VgrG proteins could potentially belong to this group: VgrG-3 of *Psy* SM (PssSM_4133) has an additional domain of unknown function (COG4253), while the C-terminus of VgrG-4 (PssSM_4495) has a weak homology to the anti-sigma factor FecI-like domain. Both proteins are well conserved among *P. syringae* isolates, and in addition, the first one is also found in *P. putida* and *X. oryzae*, while the second one is present in some *P. aeruginosa* strains. 

Another group of known T6SS effector encompasses the so-called Type VI lipase effectors (Tle), which are often encoded downstream of VgrG family proteins [103]. Among the analyzed genomes, there are six that contain genes homologous to PSPTO_5055, which is a member of the Tle3 family: A988_02933 from BRIP39023, POR16_12266 from *Por*, PssB64_01780 from *Psy* B64, PSYJA_00260/PSYJA_00265 from *Pja*, as well as unannotated genes located on contig 283 of the *Paf* genome (accession no. AWUI01000283) and on contig 6 of the *Ppa* genome (accession no. ALAC01000006). All three genes have an overlapping VgrG coding sequence upstream, as well as another ORF (Open Reading Frame) immediately downstream, which could be an immunity protein (based on the architecture of the other Tle3 gene clusters) [103]. Notably, A988_02933 contains the conventional GxSxG motif described for the family (residues 237–241), while in all other proteins, the motif is AxSxG (residues 238–242). In addition to that, the genomes of *Por* and BRIP39023 contain putative members of the Tle5 family, POR16_25160 and A988_18422, which are, however, only ~67% homologous to the described family member, PLA107_28855, from *P. syringae* pv. *lachrymans* M301315 [103]. Nevertheless, both proteins are found within the same genomic region and have a VgrG family protein encoded nearby (POR16_25135 and A988_18442, respectively). Moreover, both of them have a perfect dual HxKxxxxD motif characteristic of phospholipase D and Tle5 family members. In addition, the genome of *Ppa* contains a gene coding for a less related putative Tle5 family protein, located adjacent to one of the T6SS gene clusters (contig 27, accession no. ALAC01000027). Tle family proteins were recently shown to have antibacterial activity, and thus, they could be used by these strains to compete with epiphytic bacteria. No homologs of Tse1, Tse2 or Tse3 from *P. aeruginosa* [104] were detected in any of the Poaceae isolates.

### 2.5. Mobile and Extrachromosomal Elements

*P. syringae* genomes contain a relatively large number of mobile genetic elements (MGE) belonging to various families. The specific composition and the number of MGEs tend to differ from strain to strain [43]. More importantly, MGEs are often associated with pathogenicity islands, thus allowing their spread from one cell to another [105]. It is also known that strains have overcome race-specific resistance of their hosts after the respective avirulence gene was inactivated by an MGE insertion [106]. The heterogeneity in MGE composition is also observed for the Poaceae isolates: for example, the genome of *Psy* SM contains at least 21 transposases belonging to nine different families: ISPsy9, ISPpu10, ISPsy6, ISPpu14, ISPsy1, ISRso10, ISPsy5, ISPsy24 and IS200. In comparison, the *Psy* B64 genome encodes at least 22 transposases. The majority of these belong to the ISPsy9 and ISPpu10 families, whereas the remaining ones, with the exception of IS200, represent families not found in *Psy* SM: ISPsy7, ISPsy26, ISPsy5 and ISPsy22. In addition, the genomes encode a number of phage-type integrases/recombinases, the majority of which are truncated. A large portion of the transposable elements have at least one of their ORFs truncated, thus rendering them inactive. However, a number of transposons are present as partial sequences found on contig ends, which makes it impossible to judge their integrity. Moreover, the presence of sequence gaps only allows giving an estimate of their copy number.

Aside from simple insertional elements, the genomes also contain several prophages of variable size. While some of the prophages are very small and contain about 20 ORFs (such as prophage PSSSM-04, PssSM_4692–4712), others are over 57 kb-long (for example, prophage PSSSM-02, PssSM_2181–2256). Interestingly, prophage PSSSM-04 is the only prophage that is common to all Poaceae isolates. Prophages often serve as preferred sites of integration for other MGEs, as well as for genes acquired by horizontal transfer. One such example is prophage PSSB64-02, which has an additional ORF (PssB64_01273) present in comparison to its homolog, PSSSM-04. A more extreme example is prophage PSSSM-03, which contains a number of regions with a differing G + C profile, which is a signature of horizontal gene transfer (PssSM_4287–4288, 4292–4296, 4304–4308, 4312–4313 and 4316–4318). None of the prophages seems to be complete. However, since some components could be shared between different prophages, it is not possible to exclude that at least one is capable of forming functional viral particles. It is noteworthy that the prophage PSSSM-02 contains *recT* and *recE* genes (PssSM_2197 and PssSM_2196, respectively), which encode homologs of the lambda Red Exo/Beta proteins. The RecTE system has been successfully deployed for the recombineering of linear DNA fragments into the *P. syringae* genome [107].

Another interesting type of mobile element present in bacteria are the so-called integrative and conjugative elements (ICEs). These elements are always localized within a set of specific sites within a genome (the so-called *att* sites) and are flanked by direct repeats. They contain genes required for integration and excision and are presumably self-transmittable [108]. One of the first discovered ICEs was the *clc* element from *Pseudomonas sp*. B13, which encodes enzymes required for 3-chlorobenzoate degradation [109]. The ICEs were demonstrated to serve as vectors for the spreading of virulence factors, drug and metal resistance genes and other beneficial metabolic traits [110,111].

Several examples of ICEs were described for *P. syringae*, as well. The first one characterized was PPHGI-1 from *P. syringae* pv. *phaseolicola* 1302A [112]. This genomic region contains a gene encoding the Type III effector, *avrPhpB* (*hopAR1*), and was shown to excise itself upon infiltration into a resistant cultivar of bean. In addition to being located between two tRNA genes and having a recombinase gene required for its excision, it also contains a gene cluster for the biogenesis of conjugative pili. Interestingly, it also contains a copy of the two syringomycin biosynthesis regulator genes, *salA* and *syrF* (*salC*). A similar ICE was also identified in the genome of *Psy* B728a, where a part of it was replaced by an arsenic and copper resistance gene cluster, and in *Pto* DC3000, where only a part of the ICE is present [112]. A similar ICE was recently described for a number of *P. syringae* pv. *actinidiae* strains [6].

The genomes of all nine Poaceae isolates have two well-conserved *att* sites, each within a distinct tRNA-Lys gene. However, only seven of the genomes contain an integrated ICE (Table S3A). The exceptions are *Psy* B64 and *Paf*, where the genomic island likely has excised itself at some point after they diverged from *Pja*. Interestingly, several different types of ICEs were identified, which are located in either of the two potential integration sites: in *Ppa* and *Psy* SM, the ICE was adjacent to the *queC* gene, while in all other strains, it was located next to the *clpB* gene. The second observation was that most of the shared Poaceae isolate-specific genes were found adjacent to both *att* sequences, but not associated with the ICE. In general, among the different groups of ICEs, all structural genes were highly conserved, and the observed differences were mainly found within a variable middle part between the *pil* gene cluster and the topoisomerase III gene. The majority of the variable regions encoded heavy metal resistance genes: the ICEs of BRIP34876 and BRIP34881 contain mercury and arsenate resistance operons; *Pja* and *Ppa* have copper and arsenate resistance gene clusters, while in the case of *Psy* SM, chromate, nickel/cobalt and bacteriocin/lantibiotic efflux transporters were found. The ICE of *Por* did not contain any heavy metal resistance genes and was rather similar to the one found in pv. *actinidiae* strain NZ V-13 [6]. However, it must be noted that the respective *Por* scaffold has a number of gaps and appears to be rather misassembled, and thus, it is possible that some genes were missed. The ICE of BRIP39023, in contrast to the other six ICEs, is relatively similar to PPHGI-1. However, it contains a number of regions with no homology to PPHGI-1, namely a unique region with a number of sensor histidine kinases and transcriptional regulators (A988_21582–A988_21632), as well as another locus surrounding a poly(β-D-mannuronate)-C5-epimerase (A988_21477). Notably, the first locus encodes the T3E HopBF1 (A988_21602), while the second locus contains a truncated copy of another T3E, HopBA1-2 (A988_21482). In addition, it appears that the levansucrase C locus, which is normally located downstream of the respective *att* site, has jumped into the *syrF* homolog, resulting in its duplication. From this comparison, it appears that either there is a “population” of ICEs with distinct variable regions circulating among *P. syringae* strains, or this region is a recombination hotspot, which would also account for the observed diversity.

The last type of mobile genetic elements discussed here are plasmids. *P. syringae* strains often have one or more plasmid, some of which also encode T3Es [40,43,113], or other virulence factors, such as coronatine [8,114] or phytohormone biosynthesis enzymes [115]. Of the nine Poaceae isolates, four were identified to contain a plasmid based on the presence of a replicase gene adjacent to UV-resistance genes *rulA*/*rulB* (Table S3B). All four identified plasmids belong to the pPT32A family, which is frequently found in *P. syringae* strains [113,115]. In addition, *Psy* SM appears to have remnants of an integrated plasmid in its genome, which encodes a non-homologous replicase with a 78% similarity to the RepA protein of pPT14-32 from *P. syringae* PT14.

The identified plasmids have a complete or nearly complete Type IV-A secretion system (*virB1–virB11* and *virD4* genes [116]). No homologs of *tra*/*trb* genes from pDC3000B (accession AE016854) or *mob* genes were detected. Type IV secretion systems are frequently found on *P. syringae* plasmids; however, whether they play any role in virulence or are only involved in the spreading of the plasmids is yet to be determined [113]. Based on the current annotation, most of the proteins encoded on the detected plasmids are hypothetical, and no virulence genes were detected.

### 2.6. Other Notable Genome Components

#### 2.6.1. Defense Mechanisms against Foreign DNA

The CRISPR/Cas (clustered regularly interspaced short palindromic repeats/CRISPR-associated genes) system provides a wide-spread defense mechanism against bacteriophages. CRISPR repeat sequences contain fragments of phage DNA (the so-called spacers), which, upon transcription and processing, are involved in guiding Cas proteins, which, in turn, recognize and cleave the genome of an invading phage [117]. The system is adaptive, and new spacers can be acquired upon encountering novel invaders [118]. Among the genomes of Poaceae-colonizing strains, only *Psy* SM was identified to have two putative CRISPRs with more than one spacer sequence (CRISPR 1: 1,828,342–1,828,605; CRISPR 2: 1,828,678–1,829,372; both are inside the *inaZ* gene). However, no putative Cas proteins, which are expected to be encoded in the vicinity [119], were detected. It should be noted that *Por* and *Pja* were excluded from the search, due to a high level of genome fragmentation, which would not allow the detection of direct repeats. In addition, no PIWI-domain proteins, the analogs of the RNA interference complex protein, Argonaute [120], were identified in any of the genomes.

#### 2.6.2. Bacteriocins

Bacteriocins are ribosomally synthesized peptides, which might or might not be modified, that show bactericidal activity, usually against a narrow range of species. The producing bacterial species is unaffected by its own bacteriocins, either due to the presence of an immunity protein or by other mechanisms [121]. The most common targets of bacteriocins are the cell envelope [122], but they can also interfere with DNA, RNA and protein metabolism [121]. Among the target genomes, several bacteriocin-coding genes were identified (Table 2). From those, the product of only one gene is currently characterized. This bacteriocin is called syringacin M and was first described in *Pto* DC3000. It was shown to induce the death of susceptible bacteria by the inhibition of peptidoglycan biosynthesis through degradation of lipid II [123].

**Table 2:** Putative bacteriocin genes.

Strain	Bacteriocin	Location	Strain	Bacteriocin	Location
BRIP34876	Syringacin M	A979_09507	*Psy* SM	S-type pyocin	PssSM_0863
BRIP34881	Syringacin M	A987_14290		Colicin-DNAse	PssSM_0293 ^1^
BRIP39023	S-type pyocin	A988_14544		Bacteriocin	PssSM_4261
	Colicin-DNAse	A988_07204 ^1^	*Ppa*	Syringacin M	ALAC01000012
	Cyclized peptide	A988_02638 ^2^		S-type pyocin	ALAC01000003
*Pja*	Syringacin M	PSYJA_15707	*Psy* B64	Syringacin M	PssB64_01273
	Bacteriocin	PSYJA_00994		S-type pyocin	PssB64_04860
	Lasso peptide	PSYJA_17421	*Por*	Colicin-DNAse	POR16_04054 ^1^
	Colicin-DNAse	AEAH01000987 ^1,3^		Bacteriocin	POR16_27431
*Paf*	Syringacin M	AWUI01000310		Lasso peptide	DS996947 ^4^
	S-type pyocin	AWUI01000345			

^1^ Immunity protein-coding gene located nearby. ^2^ ORF (Open Reading Frame) located inside the specified gene on the opposite strand.^3^ The gene contains a potential frameshift. ^4^ ORF is not annotated, located between POR16_00075 and POR16_00080.

#### 2.6.3. Non-Ribosomal Peptide Synthetases/Polyketide Synthetases

Non-ribosomal peptide synthetases (NRPS) and polyketide synthases (PKS) are large multi-modular proteins with enzymatic function found in bacteria and fungi. These proteins are involved in the biosynthesis of secondary metabolites, such as antibiotics, siderophores, toxins and other bioactive compounds [124]. In *P. syringae*, with the exception of phaseolotoxin and tabtoxin, all other phytotoxins, including syringolin, are produced by NRPS/PKS-type enzymes [19,125,126]. Other NRPS/PKS gene clusters, which were found in all nine isolates, were genes for the biosynthesis of the siderophore, pyoverdine, and the surfactant, syringofactin. No yersiniabactin biosynthetic cluster was identified in any of the strains. Among NRPS/PKS genes, which were shown to be upregulated *in planta* [127], only homologs of Psyr_3722 are found in all strains, whereas a homolog of the PKS gene cluster, Psyr_4311–4315, is exclusively found in *Ppa*. As the products of these genes are unknown, it is currently not possible to draw a conclusion regarding their impact on virulence.

## 3. Experimental Section

Genomic sequences used in this study were downloaded from the NCBI (National Center for Biotechnology Information) FTP (File Transfer Protocol) server [128]. Accession numbers of the used genomic sequences are listed in Table S1. The GenBank entries for *Ppa* and *Paf* only contain nucleotide sequence data. Therefore, the genomes were re-annotated using the RAST (Rapid Annotation using Subsystem Technology) server [129] and Prokka [130]. In order to avoid any conflicts, locus_tag qualifiers were set to “panici” and “Paf”, respectively. The phylogenetic tree was generated with MEGA (Molecular Evolutionary Genetics Analysis) version 5.2.2 [131] with 1,000 bootstraps using nucleotide sequences of seven conserved genes: *gyrB*, *gap-1*, *fruK*, *pgi*, *rpoD*, *acnB* and *gltA*. Phylogenetic trees of core Type III effectors were also generated in MEGA 5.2.2 using respective protein sequences and the neighbor-joining method with 1,000 bootstraps. Ortholog cluster searches were performed with the Pan-genome analysis pipeline [132] using the MultiParanoid method with the cut off value set to 80%, an e-value of 10^−9^ and, otherwise, default parameters. Visualization of annotated genomic sequences was done using Artemis [133]. Homology searches on a gene-to-gene basis were performed using tBLASTN and BLASTP [134,135] against the non-redundant protein sequence database (nr) and other custom databases. The Type III effector sequence database was downloaded from the *Pseudomonas syringae* Genome Resources website [136]. The T3E and phytotoxin compositions of *Por* and *Pja* are based on Baltrus *et al.* [31] and Mucyn *et al.* [137], while the corresponding data for the *Pcal* strains were obtained from Sarris *et al.* [49]. Additional analysis of the *Pcal* strains was performed using IMG/ER (Integrated Microbial Genomes/Expert Review) [138]. Protein domain analysis was done using the NCBI Conserved Domain Database [139]. Bacteriocin prediction was done using BAGEL3 software [140]. The CRISPR search was performed using the CRISPRFinder tool [141].

## 4. Conclusions

Comparative genomics is a powerful tool for the discovery of virulence traits and potential host-specific adaptations. Here, we identified that *P. syringae* strains isolated from Poaceae belong to two different phylogenetic lineages, with each group evolving independently. All of these strains possess the canonical Type III secretion system, but in contrast to the majority of *P. syringae* strains, most Poaceae isolates have a relatively small repertoire of T3Es. Two strains from different phylogenetic lineages were identified to have a second T3SS similar to that of *Rhizobia*. Moreover, in line with previous studies, we have shown that strains pathogenic to the same host could have almost no overlap in T3E composition. In addition, several other putative adaptations were identified, such as a lack of quorum sensing and a number of genes that could be involved in the removal of plant anti-microbial compounds. Moreover, several traits important for leaf surface colonization, such as Type VI secretion systems and bacteriocins, are described. Lastly, the strains were compared with a non-specialized pathogen *P. cannabina* pv. *alisalensis*, which is able to grow on both dicot and monocot plants. The analyses provide a foundation for further experimental validation.

## Supplementary Files

Supplementary File 1 Supplementary Materials (ZIP, 656 KB)
