# Supplementary figures and images for: Genomics-Based Exploration of Virulence Determinants and Host-Specific Adaptations of Pseudomonas syringae Strains Isolated from Grasses
Source: Pathogens. 2014 Jan 28;3(1):121–48. doi: 10.3390/pathogens3010121 (PMC4235733; doi:10.3390/pathogens3010121)

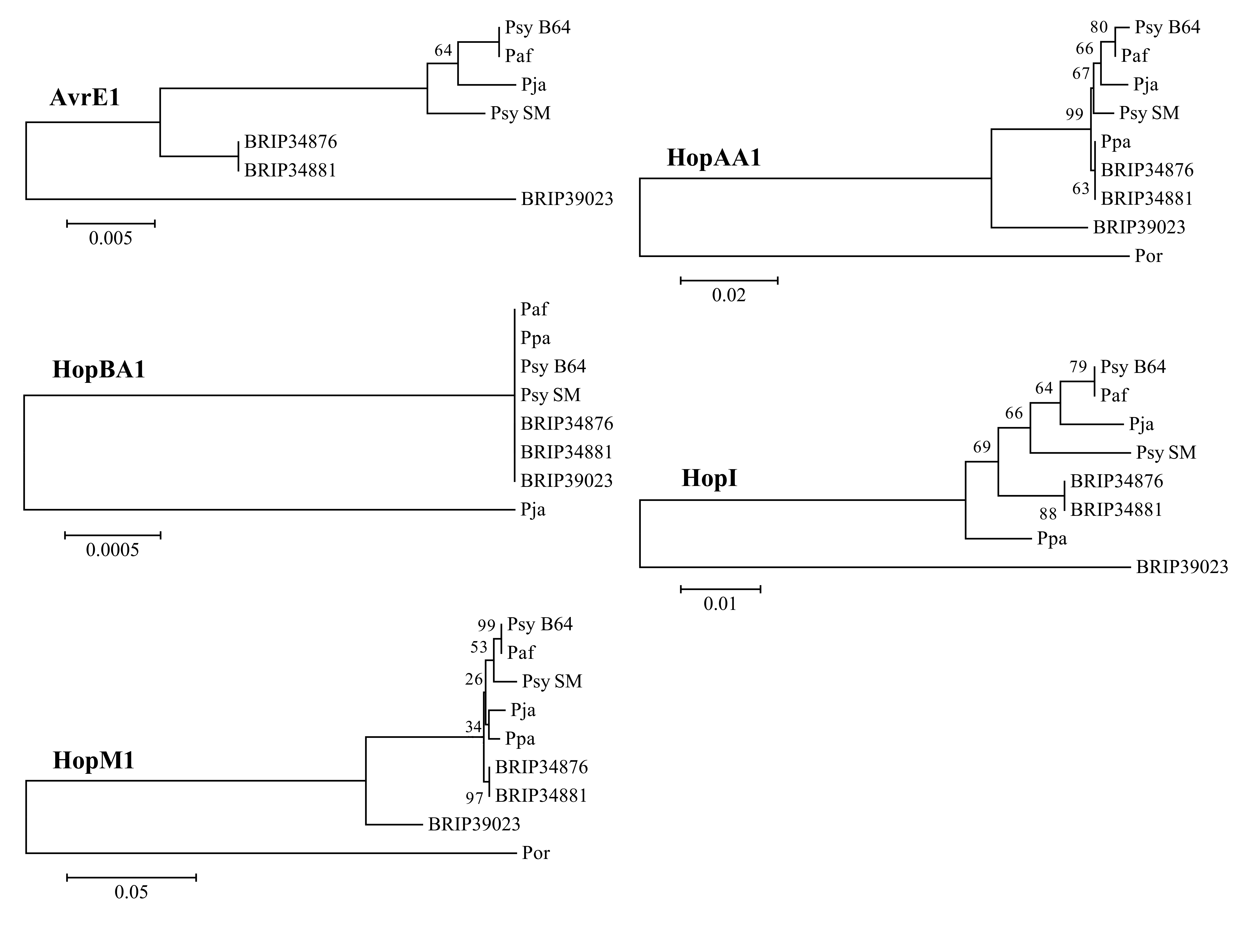

Supplement: Supplementary File 1 — Supplementary Materials (ZIP, 656 KB) [file pathogens-03-00121-s001.zip › pathogens-45676-supplementary final/Figure S1.tif]
